# Dynamic Three-Dimensional ADC Changes of Parotid Glands During Radiotherapy Predict the Salivary Secretary Function in Patients With Head and Neck Squamous Carcinoma

**DOI:** 10.3389/fonc.2021.651537

**Published:** 2021-04-13

**Authors:** Mei Feng, Qingping Yin, Jing Ren, Fei Wu, Mei Lan, He Wang, Min Wang, Lu Li, Xiaojian Chen, Jinyi Lang

**Affiliations:** ^1^ Department of Radiation Oncology, Sichuan Cancer Hospital and Institute, Sichuan Cancer Center, School of Medicine, University of Electronic Science and Technology of China, Chengdu, China; ^2^ Department of Medical Oncology, Sichuan The Third People’s Hospital, Chengdu, China; ^3^ Department of Radiation Oncology, School of Clinical Medicine, North Sichuan Medical College, Nanchong, China; ^4^ Department of Radiology, Sichuan Cancer Hospital and Institute, Sichuan Cancer Center, School of Medicine, University of Electronic Science and Technology of China, Chengdu, China; ^5^ Department of Oncology, People’s Hospital of Deyang City, Deyang, China; ^6^ Department of Radiation Oncology, Medical College of Wisconsin, Milwaukee, WI, United States

**Keywords:** diffusion-weighted imaging, radiotherapy, salivary secretary function, head and neck squamous carcinoma, parotid glands

## Abstract

**Objective:**

To investigate the changes of three-dimensional apparent diffusion coefficient (3D-ADC) of bilateral parotid glands during radiotherapy for head and neck squamous cell carcinoma (HNSCC) patients and explore the correlations with the radiation dose, volume reduction of parotid gland and the salivary secretary function.

**Materials and Methods:**

60 HNSCC were retrospectively collected in Sichuan cancer hospital. The patients were all received diffusion-weighted imaging (DWI) scan at pre-radiation, the 15^th^ radiation, the 25^th^ radiation and completion of radiation. Dynamic 3D-ADC were measured in different lobes of parotid glands (P1: deep lobe of ipsilateral; P2: superficial lobe of ipsilateral; P3: deep lobe of contralateral; P4: superficial lobe of contralateral), and the 3D-ADC of spinal cord were also recorded. Chewing stimulates test, radionuclide scan and RTOG criteria were recorded to evaluate the salivary secretary function. Pearson analysis was used to assess the correlation between 3D-ADC value, radiation dose, volume change, and salivary secretary function.

**Results:**

The mean 3D-ADC of parotid glands increased. It began to change at the 15^th^ radiation and the mostly increased in P1. However, there was no change for the maximum and minimum 3D-ADC. The 3D-ADC values of spinal cord changes were almost invisible (ratio ≤ 0.03 ± 0.01). The mean 3D-ADC was negatively correlated with the salivary secretary function (r=-0.72) and volume reduction of different lobes of parotid glands (r1=-0.64; r2=-0.61; r3=-0.57; r4=-0.49), but it was positively correlated with the delivered dose (r1 = 0.73; r2 = 0.69; r3 = 0.65; r4 = 0.78).

**Conclusion:**

Dynamic 3D-ADC changes might be a new and early indicator to predict and evaluate the secretary function of parotid glands during radiotherapy.

## Introduction

Approximate 500,000 new head and neck cancer (HNC) patients occur worldwide annually. Radiation therapy (RT), as the main non-surgical treatment, is used for over 70% patients with squamous cell carcinoma of the head and neck (HNSCC). With the development of advanced RT technology such as intensity-modulated RT (IMRT), overall survival of HNSCC patients has been improved (80% for stage I and II, 60-70% for stage III and IV) with better quality of life. However, radiation-induced xerostomia remains a common side effect and severely affects patients’ quality of life (1). Although IMRT may reduce radiation dose to the parotid glands to some extent, radiation-induced xerostomia cannot be avoided. Kam et al. (2) reported that the incidence of xerostomia was 39.3% with IMRT. A recent study reported the severe xerostomia was observed at week 7 and 8 after starting RT, and 79% of patients had grade 2 xerostomia. The percentage of patients with xerostomia dropped to 58% at follow-up month 3, 44% at month 7, 25% at month 13 and 26% at month 25 (3). Another study reported severe xerostomia was observed in patients at one month after radiation therapy and had difficulty in collecting enough amount of saliva for analysis (4). Therefore, it is important to predict the secretary function of parotid glands during radiotherapy, and it might reduce xerostomia by adjusting RT plan and/or use some particular drug in an early stage.

Several objective and subjective examinations have already been used to detect the changes in parotid function, such as salivary flowing rate, sialography, scintigraphy and salivary gland X-ray radiography (5). However, most of these tools are invasive and cannot sensitively detect the injury of parotid gland at an early stage. The increasing availability of images acquired during the delivery of RT can provide the anatomic and biological information of the patients. It was reported that radiation could induce changes of computed tomography (CT) numbers of the parotid glands based on the CTs acquired during RT for HNC (6). Very recently, researchers reported that the changes of quantitative CT textures of the parotid glands during RT delivery were correlated with xerostomia (7, 8). Magnetic resonance imaging (MRI) can provide much more details compared with CT. Functional MRI, such as diffusion weighted imaging (DWI) can reveal insight of the tissue microstructure by depicting molecular diffusion. Some studies found DWI could help to distinguish the benign tumor from malignancy tumor and there were obvious changes of DWI in primary tumor after radiation (9–11). DWI might be useful to detect early changes in the salivary glands during RT. This study was designed to acquire longitudinal MRIs during IMRT for HNSCC and to analyze the changes of spatial quantitative MRI data of parotid glands in relation to radiation dose, volume reduction and parotid function reduction.

## Materials and Methods

### Patient Selection

A total of 60 pathological confirmed head and neck squamous carcinoma patients were retrospectively enrolled from December 2016 to December 2018 in Sichuan cancer hospital. The study was approved by the ethics committee of our institution. All patients had a local disease or locoregional disease (stage I-IVa+b) according to the Union for International Cancer Control (UICC) 7th TNM staging system. The patients with the salivary gland disease and salivary cancer were excluded. Use of any medication known to affect salivary gland function was not allowed. The basic patient characteristics are listed in **Table 1**.

**Table 1:** Patient characteristics.

Items	Number	%
**Age (y)**		
<50	21	35
≥50	39	65
**Sex**		
Male	37	61.7
Female	23	38.3
**Primary site**		
Nasopharyngeal	51	85
Laryngeal	5	8.3
Oropharyngeal	4	6.7
**T stage**		
T1	2	3.3
T2	26	43.3
T3	19	31.7
T4	13	21.7
**N stage**		
N0	5	8.3
N1	20	33.3
N2	28	46.7
N3	7	11.7
**M stage**		
M0	56	93.3
M1	4	6.7
**Clinical stage**		
I	2	3.3
II	3	5
III	9	15
IV	46	76.7

### Radiotherapy Protocol

All the patients were treated with definitive image-guided IMRT (IGRT). The target volumes were outlined according to the International Commission on Radiation Units and Measurements (ICRU) 50 and 62. The prescribed doses were as follows: 70Gy to gross tumor volumes (GTVnx), 66-70Gy to positive neck lymph nodes (GTVln-R/L), 60-66Gy to high-risk clinical target volume (CTV1), 54-60Gy to low-risk clinical target volume (CTV2) and 50-54Gy to lymphatic drainage regions (CTVln). All patients were treated with 5 fractions per week in 30-33 fractions. Treatment plans were created using an inverse treatment planning system (CORVUS 3.4-4.2). The dose limits of normal organ were according to the Radiation Therapy Oncology Group protocol 0225 (RTOG0225).

### Chemotherapy

Of the 60 patients, 5 patients received radiotherapy alone, while 55 received CCRT with cisplatin 80 mg/m2 every 3 weeks for 2 to 3 cycles. Among them, 23 patients received 2 to 3 cycles of neoadjuvant chemotherapy, and 10 patients received 1 to 2 cycles of adjuvant chemotherapy. The neoadjuvant chemotherapy regimen was TPF, and the adjuvant chemotherapy regimen was cisplatin.

### MR Imaging Protocol

Anatomic MRI (T1 and T2) and DWI were acquired for each patient prior to RT, at the 15^th^ and 25th fractions, and at the completion of RT, using a 3.0T MRI scanner (Skyra, Siemens) with 20 channels of a head-and-neck combined coil. All scans extended from overhead to 2cm below the clavicle. The T1 and T2-weighted fast spin-echo images in the axial, coronal and sagittal planes were obtained before injection of contrast material. DWI sequence was performed prior to contrast injection consisted of a matrix of 160 X 160, TR 4900ms, TE_1_ 64ms, TE_2_ 103ms, b-values of 0, 500 and 800 s/mm^2^, number of excitation 1. Readout-segmented echo-planar imaging was used for DWI in our center. For both anatomical and functional imaging, transverse sequences were acquired using identical geometry to allow lesion identification and comparison at the separate time points, with a 4-mm slice thickness, 30% slice thickness as intersection gap and a field of view of 230mm X 230mm. The T1 images after intravenous injection of gadopentetate dimeglumine (0.1mmol/kg body weight Gd-DTPA, Magnevist; Bayer-Schering, Berlin, Germany) were acquired. The total acquisition time of DWI was 3 minute 42 seconds.

### Measurement of Volume and ADC Map of Parotid Gland

The parotid gland was carefully delineated by two radiation oncologists independently slice by slice. The deep and superficial lobes of both parotid glands for each patient were contoured in each MRI image. The deep and superficial lobe of parotid glands, delineated separately according to the retromandibular vein and facial nerve, were named P1: deep lobe of ipsilateral parotid gland, P2: superficial lobe of ipsilateral parotid gland, P3: deep lobe of contralateral parotid gland, and P4: superficial lobe of contralateral parotid gland. DWI data were analyzed using the Syngo MMWP (VE40D) station (Siemens Healthineers, Germany) by a radiologist and a radiation oncologist in consensus blinded to clinical and imaging characteristics. Regions of interest (ROIs, i.e., P1 to P4) were defined on the images acquired using the b-value of 0 s/mm^2^, which were automatically populated onto other b-value images by the software. ADC map was generated by using a pixel-by-pixel calculation using the equation: ADC = [ln (SI1/SI2)]/(b2– b1), where b1and b2 were gradient factors of sequences S1 and S2, and SI1 and SI2 were signal intensities by the sequences S1 and S2, respectively. The volumes of parotid glands and the maximum, mean and minimum of ADC in P1, P2, P3 and P4 were extracted automatically using the MIM software (MIM Software Inc, US). In addition, the radiation dose to each ROI was calculated from the dosimetry plan respectively.

### Measurement of Parotid Function

#### The Chewing Stimulating Test

The Saxon test is a simple, reproducible and low-cost technique to measure saliva production (12), and it was used to evaluate the saliva production at pre-RT, the 15^th^ and 25^th^ fractions and the completion of RT. The saliva production was measured by weighing a folded sterile gauze pad before and 2 minutes after chewing without swallowing. First, the sterile gauze pad and the disposable tube were weighed (S_0_). After 1 hour of prohibition and fasting, the sterile gauze pad was put into the patient’s mouth and the patient was instructed to bite the pad for 2 minutes. Then, the sterile gauze pad was removed from the patient mouth and was weighted (S1) in the test tube. The patient’s saliva (S) was calculated by formula (S=S1-S_0_).

#### Scintigraphy of Parotid Gland

Scintigraphy was acquired to evaluate the secretary function before and after RT using the Dual-head SPECT/CT γ cameras with low-energy high-resolution collimators (Siemens, Germany). At first, the patient was positioned in supine and the anterior portion of the head was imaged dynamically using a scintillation camera after a bolus intravenous injection of 370-555MBq (10 -15mCi) 99mTc-pertechnetate at 1 frame per 30 s for 30 min. Then, taking the Vitamin C, and the second scan was performed at 10 minutes after taking Vitamin C. The activity curves of both parotid glands were acquired based on the region of interest. Two parameters of the secretary function, uptake index and excretion fraction, were collected and recorded.

#### Measurement of Xerostomia

Xerostomia grade was evaluated at pre-RT, the 15^th^, 25^th^ and the completion of RT from Grade 0 to Grade 4 by the attending physician based on patient reporting using RTOG criteria as follows: G0, no change over baseline; G1, mild mouth dryness/slightly thickened saliva/may have slightly altered taste such as metallic taste; G2, moderate to complete dryness/thick, sticky saliva/markedly altered taste (i.e. copious water or other lubricants); G3, severe dry mouth, no stimulation, often need to wake up at night to drink water, and G4, acute salivary gland necrosis.

### Statistical Analysis

SPSS 20.0 was used for statistical analysis. Paired t test was used to compare the ADC values of different parotid lobes. Pearson analysis was performed on the correlation between the ADC values, delivered radiation dose, volume reduction, and secret function of the parotid glands.

## Results

### Evaluation of Salivary Gland Function

**Figure 1:**
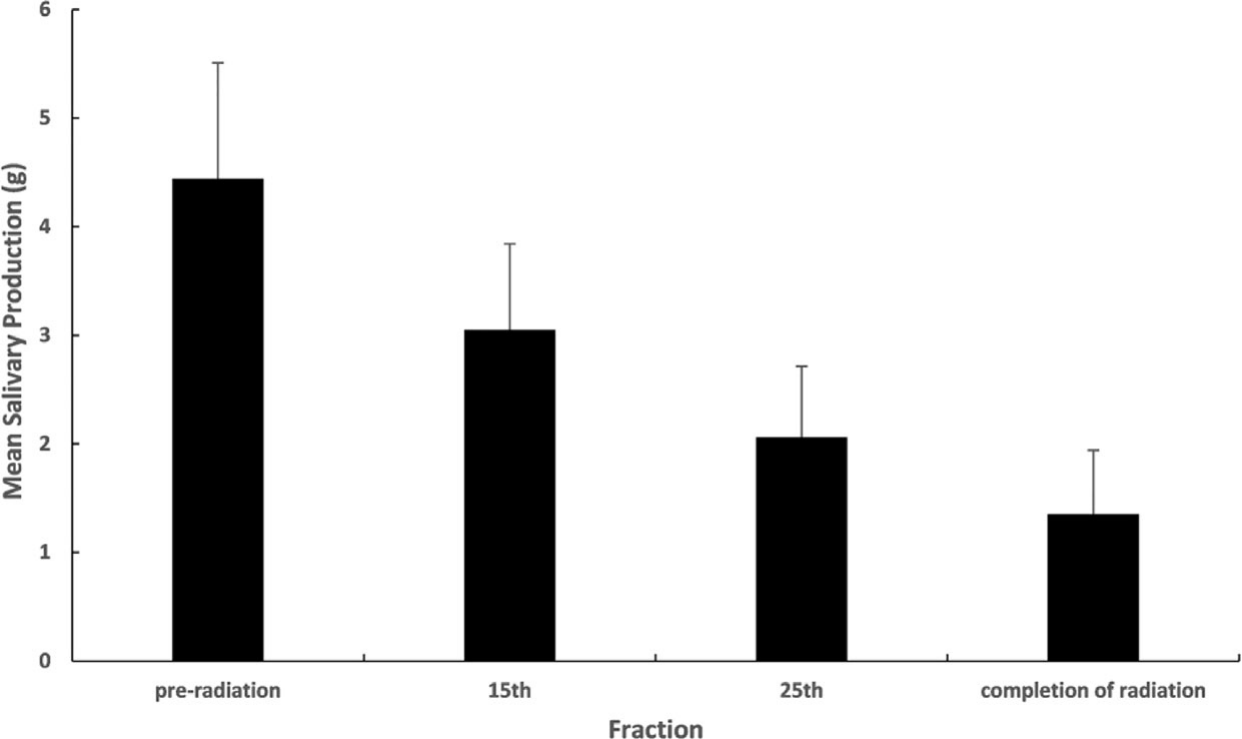
Mean salivary production during radiotherapy of chewing stimulating test.

Both chewing stimulating test and parotid gland scintigraphy showed the decreased secretion function of parotid glands after radiation for all the patients. The chewing stimulating test indicated that the mean salivary production decreased gradually at pre-RT, 15^th^ fraction, 25^th^ fraction, and the completion of RT (**Figure 1**). Similarly, the parotid gland scintigraphy showed the mean excretion fraction for ipsilateral and contralateral parotid glands reduced monotonically from 0.60 to 0.19 and 0.55 to 0.22 respectively at pre-RT and completion of RT. For the xerostomia grade, it was 10.0% for G1, 63.3% for G2, 18.3% for G3, and no G4 occurred at the completion of radiation on RTOG criteria.

### The Dynamic 3D-ADC Changes of Parotid Glands

**Figure 2:**
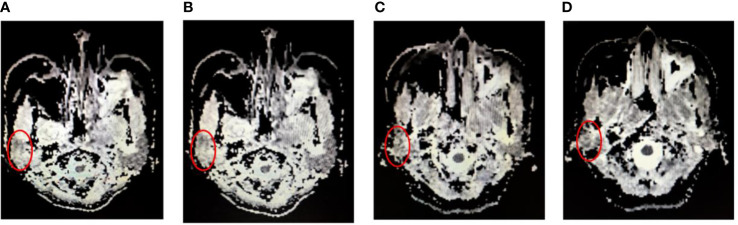
Typical DWI image for parotid glands (red circles) during radiotherapy. **(A)** at pre-radiation, **(B)** at 15^th^ radiation, **(C)** at 25^th^ radiation and **(D)** at completion of radiation.

**Figure 3:**
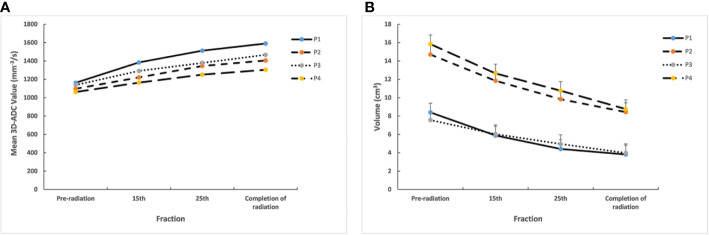
**(A)** Mean 3D-ADC changes for different lobes of parotid glands during radiotherapy. **(B)** Mean volume changes for different lobes of parotid glands during radiotherapy. P1, deep lobe of ipsilateral; P2, superficial lobe of ipsilateral; P3, deep lobe of contralateral; P4, superficial lobe of contralateral.

Typical DWI image for parotid glands during radiotherapy (**Figure 2**). All the mean 3D-ADC increases in parotid glands during RT delivery, from (1115.5 ± 109.1) x 10^-3^ mm^2^/s to (1442.0 ± 148.7) x 10^-3^ mm^2^/s, with average increased ratios of 24.8%. However, there was no obvious changes for the maximum and minimum 3D-ADC value (*P*>0.05). For the different anatomical location of parotid glands, the mean 3D-ADC value was 1163.4 ± 108.2, 1098.0 ± 151.2, 1138.1 ± 156.7 and 1062.4 ± 198.3 x 10^-3^ mm^2^/s for P1, P2, P3 and P4 respectively at pre-radiation. The mean 3D-ADC value increased dramatically with the average increased ratios of 36.7% (P1), 28.0% (P2), 28.9% (P3) and 22.8% (P4). At 15th fraction, the increased mean 3D-ADC value changed most (r=0.83). The changes of mean 3D-ADC value in spinal cord were almost invisible (≤3%) (**Figure 3A**).

### The Radiation Dose and Volume Reduction of Parotid Gland

The delivered radiation dose of the different anatomical location in parotid gland increased gradually during radiotherapy. The mean total delivered radiation dose of the parotid glands were 43.3 ± 2.9Gy (P1), 28.2 ± 1.5Gy (P2), 38.6 ± 1.9Gy (P3) and 26.2 ± 2.1Gy (P4). The mean delivered radiation dose of P1 and P3 were higher than P2 and P4 (**Table 2**). Meanwhile, the volumes of parotid glands were decreased, and the mean volume reduction was 47.3% after the completion of radiation. The mean volume reductions ratio of P1 and P3 were also larger than P2 and P4 (P1:54.5%, P2:42.6%, P3:47.4%, P4:44.6%) from pre-radiation to the completion of radiation (**Figure 3B**).

**Table 2:** Mean radiation dose for different lobes of parotid glands.

Location	Pre-radiation (Gy)	15th (Gy)	25th (Gy)	Completion of radiation (Gy)
**P1**	0	19.7 ± 1.3	32.8 ± 2.2	43.3 ± 2.9
**P2**	0	12.8 ± 0.7	21.4 ± 1.1	28.2 ± 1.5
**P3**	0	17.5 ± 0.8	29.2 ± 1.4	38.6 ± 1.9
**P4**	0	11.9 ± 1.0	19.9 ± 1.6	26.2 ± 2.1

P1, deep lobe of ipsilateral; P2, superficial lobe of ipsilateral; P3, deep lobe of contralateral; P4, superficial lobe of contralateral.

### Correlation Analysis

**Figure 4:**
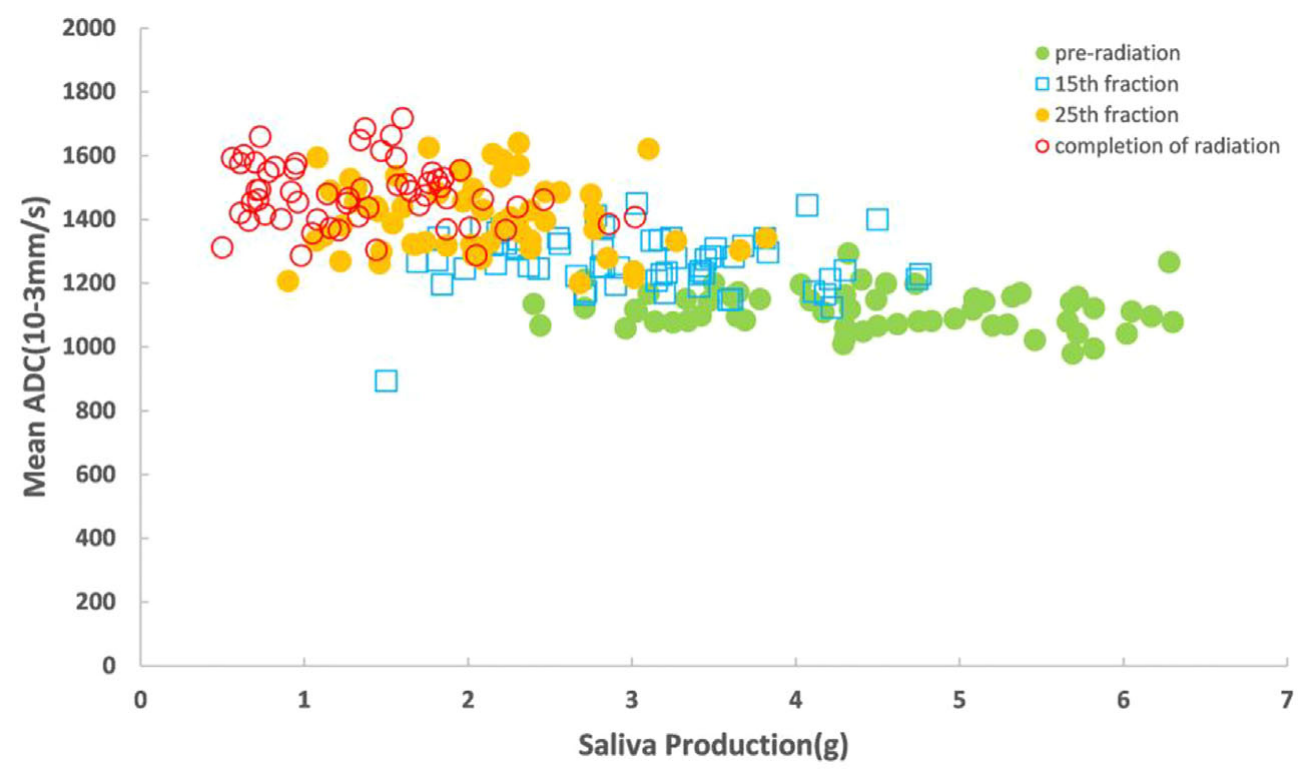
Correlation between mean 3D-ADC value for the saliva production.

**Figure 5:**
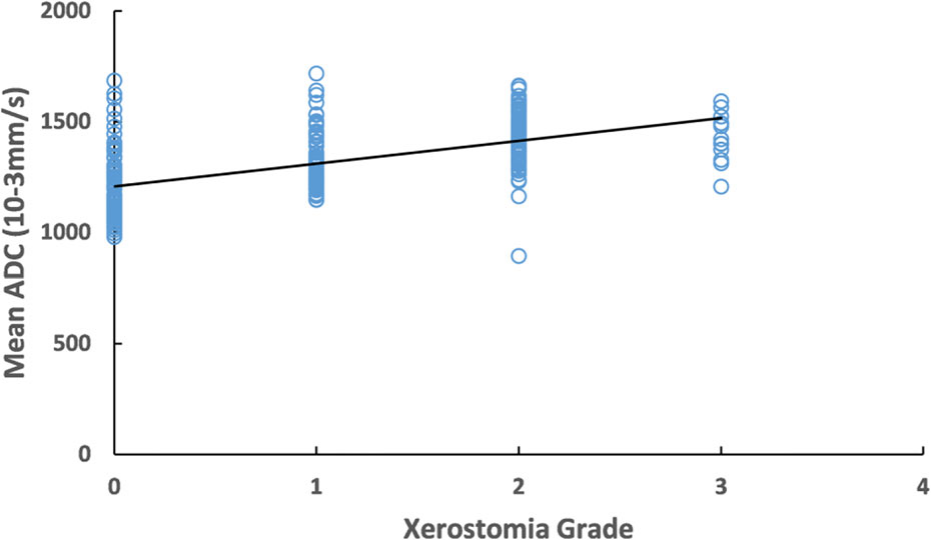
Correlation between mean 3D-ADC values for the xerostomia grade.

**Figure 6:**
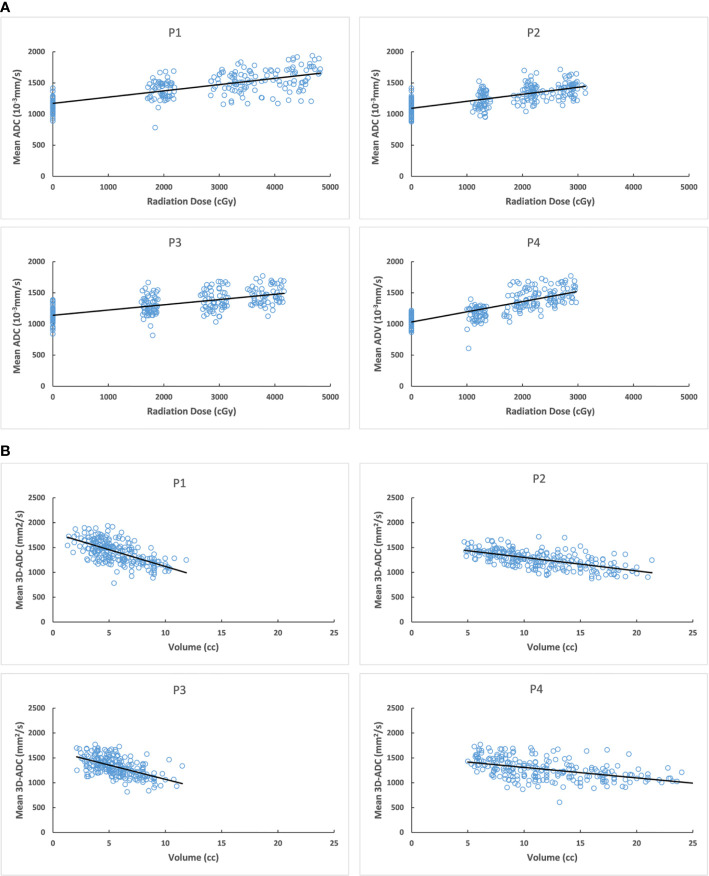
**(A)** Correlation between mean 3D-ADC changes of different parotid lobes and delivered radiation dose. **(B)** Correlation between mean 3D-ADC changes of different parotid lobes and volume reduction. P1, deep lobe of ipsilateral; P2, superficial lobe of ipsilateral; P3, deep lobe of contralateral; P4, superficial lobe of contralateral.

The increased mean 3D-ADC values during RT were positively correlated with the reduction of salivary production (r=-0.72) (**Figure 4**) and increased xerostomia grade (r=0.583) (**Figure 5**). Sub-analysis found the increased mean 3D-ADC values was positively correlated with the increased delivered radiation dose of P1, P2, P3 and P4 respectively (r1 = 0.73; r2 = 0.69; r3 = 0.65; r4 = 0.78) during RT delivery (**Figure 6A**), and it also had a significantly negative correlation with the volume reduction (r1=-0.64; r2=-0.61; r3=-0.57; r4=-0.49) (**Figure 6B**).

## Discussion

Radiotherapy is a potentially curative treatment for head and neck squamous cell carcinoma. Modern radiotherapy techniques such as IMRT could generate conformal dose distributions which allow the high radiation dose to the target volume and spare the organ at risks. One of the common and severe side effects of radiotherapy in head and neck cancer patients is the reduced saliva production, xerostomia. This complication would severely affect the quality of life for a long time. Salivary dysfunction may lead to additional effects, such as sensation of a dry mouth, altered taste, swallowing problems and speech problems which have a significant impact on the general dimensions of health-related quality of life (13).

Parotid glands are the major salivary glands that are responsible for approximately 60–65% of total saliva production (12). Sparing the parotid gland during radiotherapy could reduce the incidence and severity of xerostomia. Parotid glands would manifest both the anatomic and functional changes. For the anatomic changes, many studies had reported that the volume of parotid glands was decreased dramatically with the increased radiation dose during radiotherapy. Castadot et al. (14) showed that the volume of ipsilateral and contralateral parotid glands had a mean decrease of 0.9% and 1.0% per treatment day, respectively. Robar and colleagues (15) demonstrated that in patients subjected to IMRT, the lateral aspects of both parotid glands showed a medial translation of 0.85 mm/week, and the glands shrank by 4.9%/week. In our study, we also found that volume reduction occurred in all the lobes of parotid glands. The deep lobe received a higher dose than the superficial lobe with IMRT, therefore, the mean volume reduction of deep lobe was obviously larger than the superficial lobe from pre-RT to the completion of RT. Buettner et al. (16) found the beneficial dose-pattern analysis would minimize the dose to the lateral and cranial component of the parotid gland, and alleviated xerostomia.

With the increased delivered dose, the volume and salivary production were also decreased gradually. The volume reduction of parotid glands may have substantial correlation with the parotid gland function. Teshima et al. (12) found that the ratio of volume reduction was inversely correlated with the saliva-reduction amount in head and neck cancer patients undergoing RT. The parotid deformation may result in complex structural and functional changes in the glands leading to xerostomia during radiotherapy. In addition to age and fatty ration of parotid gland that may affect salivary production (13, 17), it was suggested radiomics would be a new biomarker to reflect the changes of irradiated tissues even in the early stage during radiotherapy (18). Our previous study found the CT numbers in parotid glands were reduced for a subset of patients and correlated with the doses received, but the correlation between CT numbers and volume reduction are weak (6). MRI quantitative analysis showed the intensity ratio of the main duct lumen to background was significantly decreased after RT when a relatively small dose was delivered to the gland. DWI is based on intravoxel incoherent motion imaging that allows visualization of molecular diffusion and microcirculation of the blood in the capillary network of biologic tissues (19). Dirix et al. (20) reported the baseline ADC value at rest was significantly higher after RT than before RT in the non-spared salivary glands but not in the spared parotid glands. Fan reported that ADC1m-post-RT for parotid gland initially increased and changed little to ADC3m-post-RT. Then, ADC6m-post-RT, ADC9m-post-RT, and ADC12m-post-RT gradually declined over time (21). There few studies reported the ADC changes of parotid glands during radiotherapy. Zhang et al. (22) reported ADC increase at 2 weeks after the beginning of RT and the amount of increase compared to baseline, and the increase rate was associated with the degree of xerostomia at 6 months after RT. However, they only observed one time point during radiotherapy and just used three adjacent sections of parotid gland to estimate the ADC, not the 3D-ADC for whole parotid gland, which may not be accurate enough for analysis. Marzi et al. (23) showed the changes of ADC at 10^th^ fraction were correlated to the volume change at the same time for the parotid glands. However, they both did not explore the correlation between xerostomia severity and ADC. These two studies were both limited to reveal the potential correlation between ADC and xerostomia in early stage of radiotherapy. In this study, we found the mean 3D-ADC value of parotid glands during radiotherapy increased with average ratios of 24.8%, and the mean 3D-ADC for deep lobe of ipsilateral parotid gland changed mostly among all the lobes. The dramatically changeable time for the mean 3D-ADC value of parotid glands was the 15^th^ fraction radiation. We also showed the increased mean 3D-ADC value was positively correlated with the delivered dose, and negatively with the volume reduction.

Till now, the mechanism of parotid gland damage and saliva reduction due to radiation is largely unknown. Wu et al. (8) revealed a higher radiation dose to the parotid gland would cause greater loss and atrophy of acinar cells, which subsequently leads to shrinkage in the gland. The atrophy of the acinar cells was also believed to be the main cause of impaired salivary secretion leading to xerostomia (24). From our study, we found that DWI changes during RT have a correlation with volume reduction and secretary function of parotid gland. This might be a new way to explore the potential mechanism.

Xerostomia is the common side effect for the HNSCC patients treated with radiotherapy. It seriously affects the quality of life for these patients. Nishi reported that a two-step IMRT with re-planning might be effective for preventing xerostomia (25). However, the timing for re-planning is controversial. One study reported that re-planning at 30Gy is essential to keep a satisfactory dose to the target volumes and avoid overdosing the organ and risks (26). Olteanu et al. (27) reported adaptive radiotherapy (ART) reduced the mean dose to parotid glands and swallowing structures by 4.6–7.1% and 3% respectively for three-phase adaptive IMRT (10^th^ and 20^th^ fractions). Image-based scoring of toxicity may offer objective instruments for “measuring” the radiation-induced damage with a strong potential in predicting individual reactions and possibility in adapting the treatment in order to reduce toxicity (28). Though we could not decide the exact re-plan timing for ART, DWI might be helpful in detecting the functional changes of parotid glands in early stage treatment, which may help to guide the optimal time for re-planning or use other medical interventions to relieve xerostomia.

In conclusion, our study indicates that the mean 3D-ADC of parotid glands increased greatly in patients with HNSCC during radiotherapy. This correlated closely with the volume reduction, salivary function and radiation dose to the parotid glands. Deep lobe of ipsilateral parotid gland might be the most damageable region for radiation. Dynamic 3D- ADC changes might be a new and early indicator to predict and evaluate the function of parotid glands, which would be help determine the timing of ART in the future. More researches are needed to explore the substantial mechanism for the image changes during radiotherapy.

## Data Availability Statement

The raw data supporting the conclusions of this article will be made available by the authors, without undue reservation.

## Ethics Statement

The studies involving human participants were reviewed and approved by Sichuan Cancer Hospital Institutional Review Board. Written informed consent for participation was not required for this study in accordance with the national legislation and the institutional requirements.

## Author Contributions

MF and JL designed the study. MF, FW, LL, and ML collected the data. JR and MW did the DWI scanning protocol. MF, QY, FW, ML and HW analyzed the data. QY and ML prepared the manuscript. MF and XC edited the manuscript. All authors contributed to the article and approved the submitted version.

## Funding

This work was supported by the Science and Technology Innovation Talent Project Funds of Sichuan Province (grant number 2020JDRC0119), Sichuan Science and Technology Department Key Research and Development Project (grant number 2020YFS0424).

## Conflict of Interest

The authors declare that the research was conducted in the absence of any commercial or financial relationships that could be construed as a potential conflict of interest.
